# Practical Medicine

**Published:** 1880-11

**Authors:** 


### Summary.

*Collaborators:*

DR. L. W. CASE, DR. R. TILLEY,
DR. BYRON W. GRIFFIN, DR. W. L. DORLAND.

### PRACTICAL MEDICINE.

DILATATION OF THE STOMACH.—(M. Sée, *Jour. de Méd.* Aug., 1880.)

Dilatation of the stomach is much more frequent than supposed, being the origin of gastric troubles which are either attributed to simple dyspepsia or to an organic lesion. It is all the more useful to discover this affection, as we have in washing out of the stomach a very efficacious means of remedying it. Among the functional symptoms of dilatation of the stomach, the most important is vomiting; it is peculiar in that when the malady has arrived to a certain degree, it is really enormous; one patient vomited twenty or twenty-five liters a day. Vomiting is sometimes followed by a state of profound collapse, by vertigo, by a real tendency to syncope. The matter vomited is of a peculiar nature, being generally mucus, and of a coffee and milk color, as is also seen in cancer; but yet this color cannot always be explained by the presence of blood mixed with this liquid. If vomiting ceases suddenly, it is usually a bad sign; it would be favorable if it coincided with a great amelioration of the general condition; but if, on the contrary, the patient remains weak, if he is not improving at the same time, the cessation of vomiting indicates that the contractility of the stomach has diminished, and that the patient no longer has the power to vomit. A frequent symptom, and one that accompanies the vomiting, is constipation. This is due to the fact that a part of the fluids, which normally should be diverted to the intestines, do not go there. Moreover, it is known that among the functions of the bile, one of the most remarkable is its causing contractions of the intestines. Now, in this case, and on account of the vomiting, only a very small quantity of the food enters the bowels, the secretion of bile is suspended, the intestinal contractions are diminished, and there follows a more or less obstinate constipation.

The appetite presents some singular variations; it diminishes or disappears even in subjects who are great eaters, and attacked with dilatation as the result of their excess; but a remarkable fact is that the appetite may return during certain periods. In those treated by washing out the stomach, this return of the appetite is speedy. In one patient of the service in particular, this phenomenon was as remarkable as the dilatation was enormous—so great that an ordinary sound of sixty centimeters in length did not reach the bottom of the stomach. And here is an important point in practice, for it may happen in certain cases, as in this one, that one does not get the desired result because the stomach is not entirely emptied by the sound; from the day when this is accomplished, an amelioration is quickly produced.

Concurrently with these local phenomena, the patient is seen to grow thinner everywhere except in the abdomen, which sometimes deceives the physician. Kussmaul has also noted this fact, that following profuse vomiting, it seems as if all the fluids of the economy disappeared, the parts become dry, and he attributes even to the peculiar state of the nervous system which results from it, the convulsions which often occur in patients of this class. An important remark, also, is that tympanitis very often exists, but that here, contrary to what is seen in ordinary cases, this tympanitis does not bring on dyspnœa. It is because here, at the same time the stomach dilates, it falls; and that in spite of the accumulation of liquid and gas, the diaphragm is not pushed up by the stomach, which is often found at a level considerably below. It is, moreover, to this tympanitis that we must attribute the pains, often very sharp, which exist in patients affected with dilatation. M. Sée believes that the pains generally called gastralgic are always due to the distention of the stomach by gases.

Dilatation of the stomach is revealed by a certain number of physical signs which confirm those already indicated. By simple inspection, for example, the prominence of the stomach is seen, but only when the disease is very much advanced. This prominence may be increased by giving the patient an effervescent powder; but one would run the risk of causing it in a healthy subject. The fluctuation, the gurgling and ballottement are signs of much more importance and which permit a certain diagnosis, especially when aided by percussion. The latter gives a dull sound at the inferior part, and a resonant sound at the superior portion when the patient is standing. Sometimes even a metallic sound is heard if there be much liquid present. A difficulty presents in certain cases; it is that the dilatation may be of the colon and not of the stomach. M. Sée has seen a patient in whom one would suppose, according to all the signs, a dilatation of the stomach existed, while the introduction of the sound into the stomach showed that it had the normal dimensions and contained no fluid. The sound is, indeed, the true means of diagnosis, for it gives certain indications by using certain precautions. After having introduced it, one may prove by aspiration the presence of an entirely abnormal quantity of fluid in the stomach. Further, one can always suppose a dilatation when the length of sound introduced exceeds sixty to seventy centimeters from the incisor teeth. Another procedure consists in searching for the extremity of the sound through the abdominal walls; but such maneuvers are dangerous, and should be absolutely proscribed. There is but one means of remedying this condition of the stomach; it is the direct washing out of the organ with the stomach-pump, or with M. Faucher's apparatus, which M. Sée has used with great success in his service. From one patient a very fœtid liquid was withdrawn, of fæcal odor and of a coffee-and-milk color, and containing gastric juice—a fact contrary to what some authors have asserted, according to whom, this substance is constantly lacking in the secretion of the stomach.

This fluid was rapidly modified by evacuation with the caoutchouc syphon and washing with alkaline water. This method might, according to M. Sée, be used in many other affections of the stomach, and is, moreover, rendered very easy by the apparatus of M. Faucher, much less dreaded and less painful for the patient than the œsophageal sound and the maneuver which it necessitates for the successive evacuation of fluid in the stomach and injection of the medicated liquid.

MEMBRANOUS ENTERITIS.—(M. Sée, *Journal de Médecine*, Aug., 1880).

The author remarked in one of his lectures, upon a peculiar state of the intestines, very often unknown to the physician, and which is manifested by the expulsion of membranous products of variable form. This has been described under the name of membranous enteritis; but this affection, associated oftenest with constipation, is much more frequent than is supposed, and if the dejecta of certain patients were examined oftener and more attentively than is usually done, one would encounter fragments resembling sometimes the white of egg, or more often still, vermicelli or the débris of teniæ. It is generally under one of these three forms that these products are found and which, upon examination, are found to consist of mucine with a little albumen and fragments of epitheleum, but not containing fibrine, that is to say the inflammatory element is lacking, which indicates that in this case the term enteritis is not justified; it would be more exact to say enterorrhœa, for the affection is very superficial. It is a state of considerable gravity, for the patients often have severe intestinal pains, and discharge sometimes voluminous masses of these products even without going to stool. There are also very often symptoms of obstruction, with considerable tympanism, and very rapid amelioration, when one can remove the obstruction, which should not be done with purgatives, as the patients do not generally support them well. M. Sée considers this condition as very frequent among women. Among them a large number of those complaining of dyspepsia are simply constipated, which is also very frequently accompanied with this membranous enteritis. That which proves clearly that in this case it is the bowel and not the stomach which suffers, is that the pains are produced a long time after the meal, and that as soon as regimen, exercise and hygiene have caused the constipation to disappear the health is re-established. Many women regarded as neuropathic, are often only in the same situation. They should be given simple laxatives, such as oil of sweet almonds or flaxseed, and regimen and exercise should be especially insisted upon.

PRUSSIC ACID AND A NEW ALKALOID IN TOBACCO-SMOKE.—Dr. Le Bon terminates with the following conclusions an elaborate paper which he has communicated to the *Journal de Thérapeutique*, September 25th, On the Existence in Tobacco-smoke of Notable Proportions of Prussic Acid, and on the Existence of a New Alkaloid:

1. The principles of tobacco-smoke, which are condensed by cooling in the mouth and lungs or in the apparatus destined to collect them, contain nicotine, earbonate of ammonia, various tarry matters, coloring substances, prussic acid combined with bases, and very odorous and very poisonous aromatic principles (*Medical Times and Gazette*). In the smoke these various substances are found mixed with a large proportion of the vapor of water and of various gaseous compounds, principally the oxide of carbon and carbonic acid.

2. The liquid resulting from the condensation of the preceding substance is endowed with extremely poisonous properties. It suffices to inject very small quantities into the circulatory system of an animal, or to cause it to be respired for some time, to induce death, after the exhibition of various signs of paralysis.

3. The properties of tobacco-smoke, which up to the present time have been attributed solely to nicotine, are also due to prussic acid and to various aromatic principles, especially an alkaloid, collidine. This is a liquid body of an agreeable and very penetrating odor, the presence of which had been exhibited in the distillation of various organic matters, but the physiological properties of which were entirely unknown. It contributes in great part to giving its odor to tobacco-smoke, and so penetrating is its perfume that but a single drop suffices to impart a very strong odor to a large quantity of water.

4. Collidine is an alkaloid as poisonous as nicotine. The twentieth part of a drop kills a frog rapidly, producing symptoms of paralysis. Only a few instants' breathing it, induce muscular debility and vertigo.

5. It is to the presence of prussic acid and the various aromatic principles that several phenomena are due, such as vertigo, pain in the head and nausea, which are produced by certain tobaccos either poor in nicotine or destitute of it, while other tobaccos rich in nicotine do not produce any analogous effects.

6. The proportion of prussic acid and aromatic principles contained in tobacco smoke varies in different tobaccos, those of Havana and the Levant containing the strongest doses.

7. The black semi-fluid matter which condenses in the interior of pipes and cigar-holders contains all the substances enumerated, and especially large quantities of nicotine. It is extraordinarily poisonous, two or three drops sufficing to kill an animal of small size.

8. The combustion of tobacco destroys only a small part of the nicotine which it contains, so that this is found in great part in the smoke. The proportion susceptible of being absorbed by smokers, and which we have determined in our experiments, varies according to the conditions in which these latter are placed. It is scarcely ever less than fifty centigrams in each hundred grams of tobacco smoked. The quantity of ammonia absorbed at the same time is about equal.

9. Of the different modes of smoking, that in which the amount of nicotine and the various other principles absorbed is greatest, is smoking, so that the smoke is respired; that in which the proportion is least, is smoking the narghil or pipe with a long tube in the open air without respiring the smoke.

10. Nicotine kills animals instantly in doses of two or three drops, but in infinitely smaller doses it causes paralysis and death. A frog introduced into a vessel containing an aqueous solution of nicotine at $\frac{1}{200000}$, or about one drop to a liter of water, succumbs in some hours. The same occurs if the frog be placed under a funnel containing a single drop of nicotine in a roll of cotton wool. The vapor disengaged from nicotine while boiling kills animals instantly without leaving them time to move.

11. Tobacco smoke contains about eight liters of oxide of carbon per hundred grams of tobacco burned. Our experiments prove that it is not to this gas that it owes its poisonous properties.

12. Among the most certain effects which the smoke of tobacco determines in the long run in man, may be mentioned visual disturbances, palpitations, tendency to vertigo, and especially diminution of memory.—*Louisville Medical News.*

THE REGULATION OF BODY HEAT.

A Fränkel, in *Centralblatt für die Med. Wissenschaften,* endeavors to solve the question, in what way and through what means, during changes in the production of heat in the body, the changes in the cutaneous circulation which make up the compensation through the giving off of heat, are effected. Heidenhain—as is well known—was the first to prove that the heat of the body is affected by the system of nerves under the sole agency of the circulation, by showing that, under reflex as well as indirect irritation of the medulla oblongata, the temperature of the blood in the large vessels decreases. The acceleration of the blood stream, observed by Heidenhain, has been traced by Ostroumoff to an irritation of the nerve-fibers which dilate the vessels. Over-production of heat in the organism under normal conditions, is dependent upon strong muscular activity and increased food absorption, both of which go hand in hand with increase of production of carbonic acid. The carbonic acid, however, directly irritates the centers located in the medulla oblongata, and consequently the formation of this end-product of the change of matter must be also connected with regulation of heat. As the accumulation of carbonic acid coincides with deficiency of oxygen, the author tried to decide his task by insufflation of differently combined quantities of carbonic acid, oxygen and nitrogen into the lungs of dogs. The insufflation was done with a compressible India-rubber ball, which was connected by a three-branched tube canula, tied into the trachea of the animal, with gas reservoirs, and with the atmospheric air. The cutaneous temperament of the paws and that of the rectum were measured. Some days before carrying out these experiments one sciatic nerve was cut through, in order to ascertain, by comparative measurements of both hind-paws, whether the changes in the cutaneous temperature, eventually to be observed under the influence of insufflation, originated from a changed innervation of the vessels remaining in connection with the centers or not. Three series of experiments showed, on the side on which the sciatic nerve was uncut, a rise of temperature; on the paralyzed side a standstill, or even fall of temperature. The inconstancy of the influence of the accumulation of carbonic acid on the irritation of the nerves dilating the vessels, observed by the author in other experiments, he traces back to prolonged restraint of the animals, and the simultaneous decrease of the temperature inside the body, and the continuous influence of the curare. The author is now of opinion that the increased production of carbonic acid in the organism, which occurs in increased temperatures under the influence of muscular activity, as well as of absorption—absorption of food—is one of the factors which regulate the activity of the nervous apparatus that governs the extent of the production of heat.

